# BMScan: using whole genome similarity to rapidly and accurately identify bacterial meningitis causing species

**DOI:** 10.1186/s12879-018-3324-1

**Published:** 2018-08-15

**Authors:** Nadav Topaz, Dave Boxrud, Adam C. Retchless, Megan Nichols, How-Yi Chang, Fang Hu, Xin Wang

**Affiliations:** 1 Meningitis and Vaccine Preventable Diseases Branch, Division of Bacterial Diseases, National Center for Immunization and Respiratory Diseases, Office of Infectious Diseases, Centers for Disease Control and Prevention, 1600 Clifton Rd NE, MS D-11, Atlanta, GA 30329 USA; 2 Minnesota Department of Health Public Health Laboratory, St. Paul, MN USA

**Keywords:** Bacterial meningitis, Comparative genomics, Species identification, Reference collection

## Abstract

**Background:**

Bacterial meningitis is a life-threatening infection that remains a public health concern. Bacterial meningitis is commonly caused by the following species: *Neisseria meningitidis, Streptococcus pneumoniae*, *Listeria monocytogenes*, *Haemophilus influenzae* and *Escherichia coli*. Here, we describe BMScan (Bacterial Meningitis Scan), a whole-genome analysis tool for the species identification of bacterial meningitis-causing and closely-related pathogens, an essential step for case management and disease surveillance. BMScan relies on a reference collection that contains genomes for 17 focal species to scan against to identify a given species. We established this reference collection by supplementing publically available genomes from RefSeq with genomes from the isolate collections of the Centers for Disease Control Bacterial Meningitis Laboratory and the Minnesota Department of Health Public Health Laboratory, and then filtered them down to a representative set of genomes which capture the diversity for each species. Using this reference collection, we evaluated two genomic comparison algorithms, Mash and Average Nucleotide Identity, for their ability to accurately and rapidly identify our focal species.

**Results:**

We found that the results of Mash were strongly correlated with the results of ANI for species identification, while providing a significant reduction in run-time. This drastic difference in run-time enabled the rapid scanning of large reference genome collections, which, when combined with species-specific threshold values, facilitated the development of BMScan. Using a validation set of 15,503 genomes of our species of interest, BMScan accurately identified 99.97% of the species within 16 min 47 s.

**Conclusions:**

Identification of the bacterial meningitis pathogenic species is a critical step for case confirmation and further strain characterization. BMScan employs species-specific thresholds for previously-validated, genome-wide similarity statistics compiled from a curated reference genome collection to rapidly and accurately identify the species of uncharacterized bacterial meningitis pathogens and closely related pathogens. BMScan will facilitate the transition in public health laboratories from traditional phenotypic detection methods to whole genome sequencing based methods for species identification.

**Electronic supplementary material:**

The online version of this article (10.1186/s12879-018-3324-1) contains supplementary material, which is available to authorized users.

## Background

With an estimated 1.2 million cases occurring globally every year, bacterial meningitis is life-threatening infection that remains a public health concern [1]. Numerous pathogens can cause bacterial meningitis, and the case-fatality rate and prevalence of the disease per pathogen varies by region, country and age group [2]. Bacterial meningitis is commonly caused by *Neisseria meningitidis, Streptococcus pneumoniae*, *Listeria monocytogenes*, *Haemophilus influenzae* and *Escherichia coli* [3]. Identification of the meningitis-causing bacterial species is a critical step for case management and disease surveillance.

A long-standing standard in species identification is DNA-DNA hybridization (DDH), a technique that relies on the sequence similarity between pools of DNA to calculate distances between two organisms. With DDH, the traditional cutoff value for same-species comparisons was determined to be 70% [4]. Due to the complexity of this method, laboratory methods targeting bacterial phenotypic features have been developed for species determination. Multiple phenotypic methods are often required in order to confirm a particular species.

As generating genomic data has become more accessible, whole-genome sequencing (WGS) based tools have been developed that allow for comparison against reference collections of representative genomes for species identification, with the additional benefit of having the genome collection available for further downstream analyses. One of these WGS-based comparative tools is Average Nucleotide Identity (ANI) [5], which assesses genomic similarity by comparing homologous nucleotide fragments between two genomes. ANI has been considered to be the gold standard whole-genome method for prokaryotic species identification [6]. An ANI of 95% has been reported to be comparable to the 70% DDH threshold value for species delineation [7]. Two common implementations of ANI are ANI BLAST (ANIb) and ANI MUMmer (ANIm), which use the BLAST algorithm [8] and the MUMmer method [9] respectively. While these ANI methods provide a high-level of resolution for assessing genetic similarity between genomes, the trade-off is their long computational run-time, rendering them unfeasible for scanning multiple genomes against large reference collections.

To address this limitation, tools focused on improving run-time by using k-mer-based comparisons for estimating genetic distances were developed. A main example of these tools is Mash [10], which applies the MinHash algorithm to estimate the distance between two genomes. In their paper describing Mash, Ondov et al. showed that the genetic distance estimated by Mash is strongly correlated to approximately 1-ANI, such that a Mash distance of 0.05 corresponds to an ANI of 0.95.

We sought to test whether Mash could provide a resolution for species delineation that was equivalent to that provided by ANI for our focal species. We then used Mash to establish species-specific threshold values for each of our focal species. Finally, we validated the accuracy of these threshold values using a new collection of genomes consisting of our focal species, as well as several closely-related and sister-species. Using these results, we have developed BMScan, a program that rapidly and accurately assigns uncharacterized isolates to our focal species when genome-wide similarity is above the curated, species-specific threshold values.

## Methods and implementation

### Establishing reference collection

The first step in this process was to establish a reference collection of genomes which, in their entirety, capture the diversity within each individual species. This process entailed gathering genomes for each of our 17 focal species, identifying the representative genomes from each species, and subsequently testing these representatives to ensure that they capture the diversity within their respective species.

The following species of interest were selected due to their reported ability to cause bacterial meningitis: *Neisseria meningitidis, Haemophilus influenzae, Streptococcus pneumoniae, Listeria monocytogenes, and Escherichia coli*. In addition to these five species, we chose to include several additional species of interest from the genera *Neisseria* and *Haemophilus*. These additional species were: *Neisseria cinerea, Neisseria elongata, Neisseria lactamica, Neisseria gonorrhoeae, Neisseria subflava, Neisseria mucosa, Neisseria weaveri, Neisseria polysaccharea, Haemophilus haemolyticus, Haemophilus parainfluenzae, Haemophilus parahaemolyticus,* and “*Neisseria bergeri.” Neisseria bergeri* has historically been characterized as a variant of *Neisseria polysaccharea,* but has since been suggested to be reclassified as a novel species [11]. We followed the proposed reclassification of Neisseria species by Maiden et al. [12], classifying *Neisseria flavescens* as subspecies of *Neisseria subflava,* and *Neisseria sicca* and *Neisseria macacae* as subspecies of *Neisseria mucosa*. Notably, the methods described here for generating the reference collection for BMScan are not unique for our chosen focal species, and can be applied to any other bacterial species of interest to the user.

The genomes for each of these species were obtained from three main sources: 1) the Bacterial Meningitis Laboratory (BML) isolate collection, 2) NCBI’s RefSeq [13], and 3) the Minnesota Department of Health Public Health Laboratory (MDH) isolate collection. All of the isolates from the BML collection were tested for their respective species through a combination of biochemical and molecular testing, including the API NH strip system [14] and PCR for species-specific genes [15]. The isolates from the MDH collection were tested for their respective species through a series of biochemical tests, such as rapid sugars, slide agglutination and other classical microbiological methods [16].

All isolates from CDC BML and MDH were sequenced using either Illumina (*n* = 1782) or Pacific Biosciences (PacBio) technologies (*n* = 82) as described previously [17]. These Illumina reads were assembled using SPAdes version 3 [18], and the PacBio reads were assembled using PacBio’s Hierarchical Genome Assembly Process version 3 (HGAP) [19]. The collection was supplemented with additional assemblies from NCBI’s Refseq (*n* = 820) and then processed through the dRep pipeline [20]. The dRep software consists of a set of command-line tools for clustering a given set of genomes and identifying high-quality representative genomes for each cluster. This pipeline analyzes the entire set of genomes, performs a rapid, primary-clustering with Mash using a threshold of 0.90, followed by a slower, secondary-clustering within each primary-cluster with ANI using a threshold of 0.995. After clustering is complete, dRep identifies the representative genome from within each cluster. These representative genomes.

The representative genome is selected through a scoring process, whereby each genome is scored according to a formula which factors several components of genome quality, such as: 1) genome completeness, 2) N50, 3) contamination, 4) genome size and 5) strain heterogeneity. These quality metrics were determined using the CheckM [21] module within dRep. The highest scoring genome for each cluster was selected as the representative of the respective cluster, and these representatives were then compiled to create our reference collection. Each representative genome is either a divergent strain that was selected from a cluster of one by being less than 99.5% similar to any other genome in the collection, or is the best quality genome from a cluster of highly similar genomes which are at least 99.5% similar to other genomes within that cluster. Ultimately, this workflow ensures that each genome in the reference collection both captures the diversity of its respective cluster and is of high quality.

### Obtaining threshold values for each species

After finalizing the reference collection, threshold values were established for each of the 17 focal species through all-vs-all pairwise comparisons using both ANI and Mash. The results of these comparisons were parsed, and the Mash distances were converted into Mash scores (1-Mash distance) to be easily comparable with ANI, such that a lower Mash score indicates a greater distance between two genomes. The smallest score between members of the same species was recorded as that given species’ threshold value. The threshold values for both ANI and Mash serve as indicators of confidence for each method that an unknown genome belongs to that given species, as these thresholds mark the greatest distance between two members of the same species within the reference collection.

The final threshold values for each species were stored in a SQL database. In addition to these values, this database also contains meta-data for each genome, such as its source location, file name, ID, genus and species. The database SQL schema is provided as supplement (Additional file 1).

The results of each all-vs-all Mash and ANI comparison were tested for linear correlation using Pearson’s correlation coefficient test. For this analysis, we only included pairwise comparison values of 0.90 or above, as we were primarily interested in the correlation between Mash and ANI for intra-species comparisons and for identifying inter-species boundaries. To assess the agreement between ANI and Mash values above 0.90, we created a Bland Altman plot [22].

### Species delineation with Mash

In combination with the species-specific threshold values, we sought to represent Mash’s ability to delineate between bacterial species within the same genus. For this analysis, we ran Mash on the *Neisseria* and *Haemophilus* genomes within the reference collection, and used those results to generate neighbor-joining [23] trees. These trees were generated by formatting the Mash results as distance matrices, parsing each matrix and creating the tree with a custom python script built with the BioPython [24] and Phylo [25] packages. The final trees were visualized using the iTOL [26] package.

### Development of BMScan

BMScan was developed to serve as a comprehensive program for bacterial meningitis species identification. The tool was written using the Python programming language (https://www.python.org/). The program is a pipeline with Mash at its core, which coordinates with a custom-built SQL threshold database for rapid retrieval of the results. The code for BMScan can be found here https://bitbucket.org/ntopaz/bmscan.

### Assessing performance of BMScan

In order to check whether these threshold values confidently capture the diversity within each species, we established a validation set. This process consisted of downloading all available genomes for each species of interest from both RefSeq and Genbank [27], as well as the genomes for additional closely-related and sister species of those included in the reference collection (*N* = 15,503). These additional species include *Streptococcus mitis, Escherichia albertii, Escherichia fergusonii,* and *Listeria innocua.* Several *Neisseria* species had very few (less than or equal to 10) available genomes on NCBI, so these species were supplemented with genomes from the *Neisseria* isolate collection on PubMLST [28] (*N* = 208). Each of the genomes in the set was scanned using BMScan, and the results were analyzed to assess the performance of the tool. As NCBI’s RefSeq is one of the main sources of genomes for the reference collection, any pairwise comparisons between identical genomes were filtered out.

## Results

### Establishing threshold for each species

The initial reference set prior to the dRep process consisted of a total of 2677 genomes. After being processed through dRep, 759 genomes remained, a reduction of 71.65%. The counts for each species before and after dRep are shown in Table 1. Additional meta-data for the 759 reference collection genomes is available as supplement (Additional file 2). *Neisseria gonorrhoeae* had the largest change in number of genomes, reducing 400 genomes down to 31, a change of 92.25%, while *Neisseria weaveri* had the smallest, with no change in the number of genomes.

**Table 1:** Genome counts before and after dRep

Species	Pre-dRep	Post-dRep	% Change
Neisseria gonorrhoeae	400	31	92.25%
Haemophilus influenzae	948	117	87.66%
*Listeria monocytogenes*	58	21	63.79%
*Escherichia coli*	151	60	60.26%
Neisseria meningitidis	859	352	59.02%
Neisseria polysaccharea	6	3	50.00%
Haemophilus parainfluenzae	35	18	48.57%
Haemophilus haemolyticus	66	37	43.94%
Neisseria elongata	11	7	36.36%
Neisseria lactamica	47	31	34.04%
Streptococcus pneumoniae	33	22	33.33%
Neisseria cinerea	6	4	33.33%
Haemophilus parahaemolyticus	5	4	20.00%
Neisseria bergeri	5	4	20.00%
Neisseria subflava	39	34	12.82%
Neisseria mucosa	8	7	12.50%
Neisseria weaveri	7	7	0.00%
Total	2684	759	71.65%

These 759 representative genomes were combined into a reference collection and used for comparing Mash, ANIm and ANIb. Each test consisted of running all-vs-all comparisons for each of the 759 genomes in the reference collection, resulting in a total of 576,081 pairwise comparisons for each method. The run-time of these comparisons using 40 CPU threads is reported in Table 2. ANIb had the longest run-time, taking 75.2 h to complete the comparisons. ANIm took 17.2 h to complete the comparisons, a significant reduction in run-time compared to ANIb. Finally, Mash took a fraction of the time of the two ANI methods, completing all comparisons in 12.7 s.

**Table 2:** Run-Time of ANI methods vs Mash

Method	Run-Time of All vs All Comparisons		
	Seconds	Minutes	Hours
ANI BLAST	358,625	5977.1	99.6
ANI MUMmer	62,127	1035.4	17.2
Mash	12.7	0.21	.003

The thresholds for both ANI and Mash were obtained through parsing the results of each all-vs-all comparisons and identifying the lowest ANI value and Mash score respectively for each species. Threshold values obtained for both ANI methods and Mash are shown in Table 3. The differences in threshold values between the methods are caused by the varying algorithms used by each method for determining genetic similarity.

**Table 3:** Species-specific threshold values and differences for each method

Species	Threshold Values		
	ANI MUMmer	ANI BLAST	MASH Score
Neisseria meningitidis	0.965	0.964	0.975
Neisseria gonorrhoeae	0.989	0.992	0.993
Neisseria lactamica	0.968	0.967	0.974
Neisseria cinerea	0.967	0.967	0.97
Neisseria elongata	0.963	0.961	0.961
Neisseria mucosa	0.961	0.959	0.964
Neisseria subflava	0.945	0.943	0.945
Neisseria weaveri	0.989	0.988	0.99
Neisseria bergeri	0.957	0.955	0.97
Neisseria polysaccharea	0.95	0.947	0.957
Haemophilus influenzae	0.947	0.943	0.95
Haemophilus parainfluenzae	0.933	0.93	0.934
Haemophilus haemolyticus	0.944	0.94	0.946
Haemophilus parahaemolyticus	0.953	0.952	0.963
Escherichia coli	0.968	0.962	0.962
Listeria monocytogenes	0.936	0.932	0.946
Streptococcus pneumoniae	0.976	0.974	0.978

**Fig. 1:**
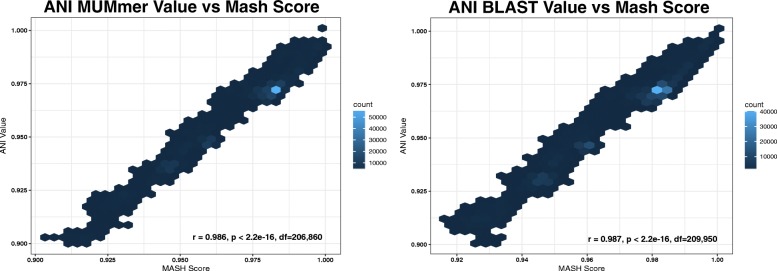
Correlation between ANI methods and Mash: The figure above shows the linear correlation between both ANI methods and Mash. The relationship between Mash and ANI MUMmer is shown on the left and ANI BLAST on the right. In both cases, there was a strong linear correlation between the methods for all values above 0.90

The linear correlation of the results from both ANI methods and Mash are shown in Fig. 1. These results show that there is a very high level of correlation between the results of each method (ANIm vs Mash: *r* = .986, *p* < 2.2 × 10^− 16^, df = 206,860; ANIb vs Mash: *r* = .987, *p* < 2.2 × 10^− 16^, df = 209,950) for values above 0.90. The strong correlation between the methods suggests that for values above 0.90, Mash provides a similar level of resolution for species delineation to that of ANI, and all threshold values obtained by both ANI and Mash were above 0.90.

**Fig. 2:**
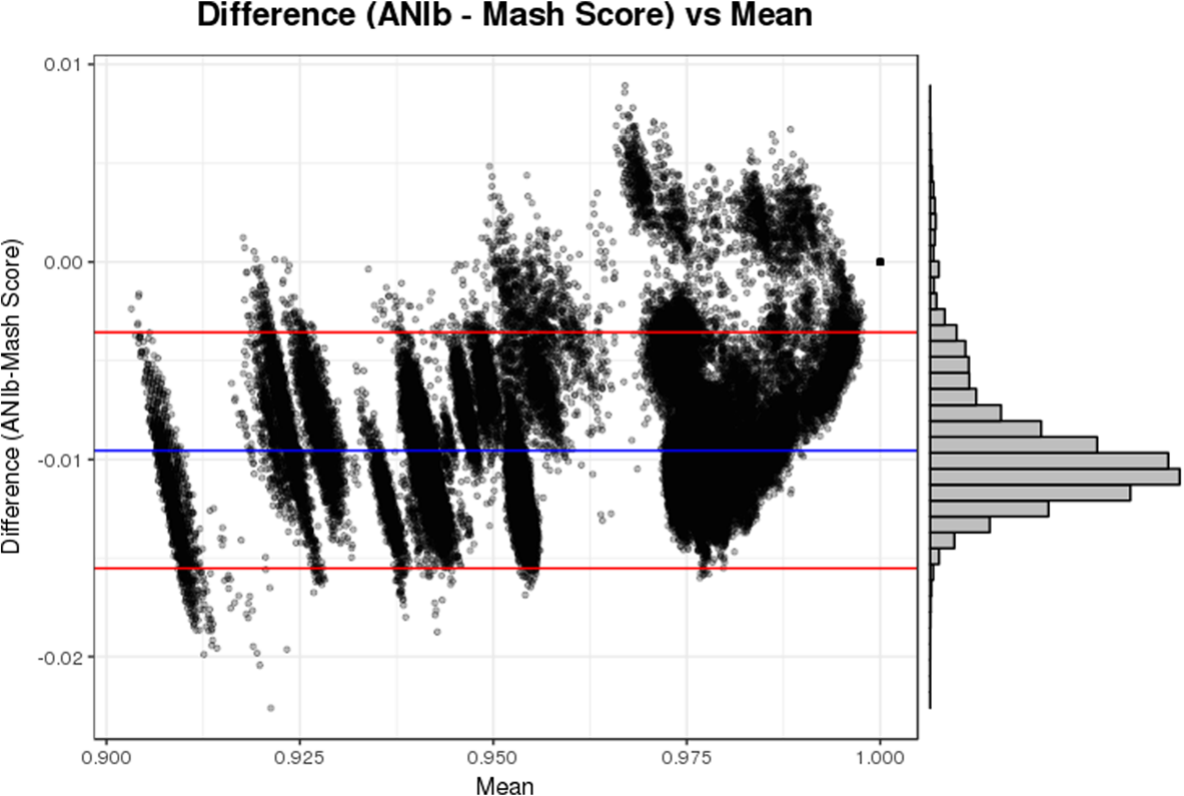
Bland Altman Plot for ANIb and Mash: The Bland Altman plot above shows the differences between ANIb and Mash score values above 0.90 (Y axis) plotted against the mean (X axis). The blue line indicates the mean of the differences and the red lines indicate the upper and lower limits of agreement (LoA). These limits of agreements were calculated by taking 1.96 * standard deviation of the differences and adding/subtracting the value from the mean. The value for the mean of the differences is − 0.00954, the upper LoA is − 0.00357, and the lower LoA is − 0.01552

The agreement between ANI and Mash was assessed using a Bland Altman plot. Figure 2 shows the plot for ANIb and Mash Score values above 0.90. In total, 209,955 points are represented in the figure, and 199,526 (95%) fall within the calculated limits of agreement. It is interesting to note that the difference between ANIb and Mash seems to get more positive as the value approaches 1, while being more negative for values closer to 0.90. The Bland Altman plot for ANIm and Mash provided similar results and is available as supplement (Additional file 3).

### Delineation of *Neisseria* and *Haemophilus* species

**Fig. 3:**
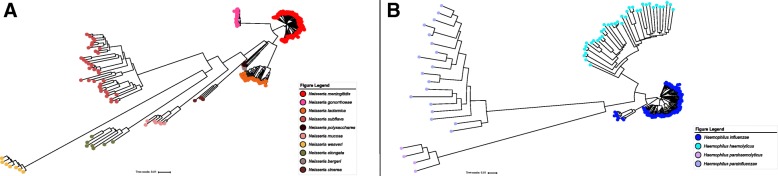
Phylogeny using Mash distances: A neighbor-joining tree was constructed using the results of the Mash pairwise comparisons. These trees represent the resolution provided by Mash for species delineation for *Neisseria* and *Haemophilus*. Each species is color-coded, and the colors for each species is listed in the figure legends provided. **a** This tree consists of all 10 *Neisseria* species in the reference collection. A total of 480 isolates are represented on this tree. **b** This tree consists of the 4 *Haemophilus* species. A total of 176 isolates are represented on this tree

In order to assess Mash’s ability to delineate between our two major genera, *Neisseria* and *Haemophilus*, we used the representative genomes for each of these genera to generate phylogenetic trees. These trees are shown in Fig. 3. The left panel contains all 10 *Neisseria* species within the reference collection, while the right panel consists of the 4 *Haemophilus* species.

Mash was able to accurately delineate between all of the *Neisseria* and *Haemophilus* species. The *Neisseria* species form compact, yet distinct clusters. *Neisseria polysaccharea* and *Neisseria bergeri* form two distinct, closely-related clusters, consistent with the reclassification of *Neisseria bergeri* from a subvariant of *Neisseria polysaccharea* to its own species.

Overall, the resolution of species delineation provided by Mash, combined with the drastic difference in run-time and strong correlation between both ANI methods resulted in our choice to use Mash in BMScan.

### BMScan workflow

**Fig. 4:**
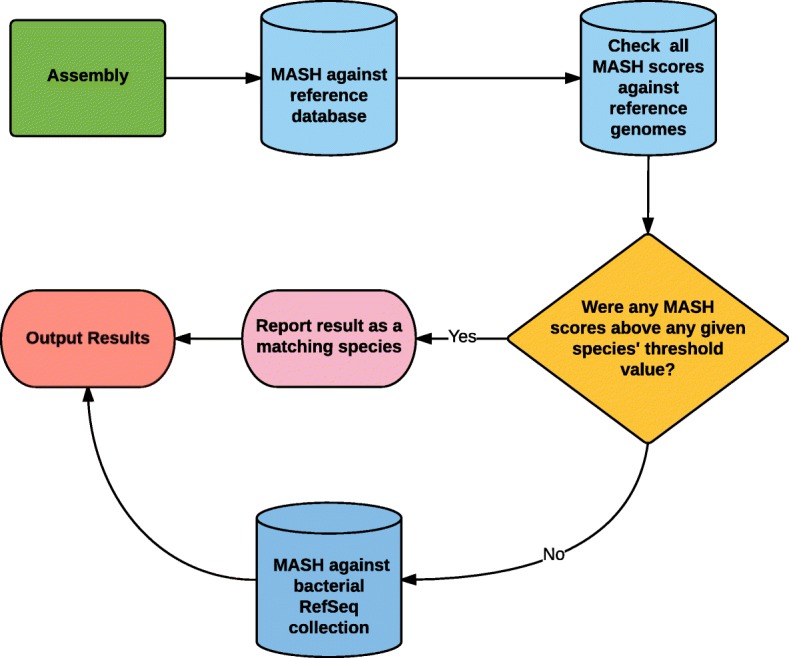
BMScan Workflow: The figure above shows the workflow for a query in BMScan. The input query is an assembly file in FASTA format. The query gets scanned against the reference collection using Mash. The species and threshold value for each hit is obtained from the SQL database component. If the Mash score of the pairwise comparison was above the threshold for that species, it is considered a high confidence result and is stored for output. If none of the hits were above the threshold values, that query is scanned against the bacterial RefSeq collection with Mash. The top hit from this step is stored for output, and a flag is added noting that this is query did not produce any results that were above the threshold values for our set of species

The BMScan workflow (Fig. 4) consists of a systematic approach for identifying the species of the query. This workflow consists of an iterative two-step process: 1) scan query against reference collection and compare with established thresholds for each species and 2) compile below-threshold scoring queries and scan these queries against the RefSeq bacterial database as an exploratory search.

The input for BMScan is a genome assembly in FASTA format. The user can specify either a directory of assemblies or a file listing the paths to multiple assemblies. Each assembly is then sketched with Mash and compared against the precomputed sketches of the reference collection. The resulting distance matrix is then parsed, and the result of each pairwise-comparison is compared against the SQL database to retrieve threshold and species information. If the result of the pairwise comparison is equal to or greater than the hit’s threshold value, it is stored as a result. This step serves to continuously assess the threshold values by always considering all of the hits per query.

If no above-threshold scores are found for the query, it is then scanned against the bacterial RefSeq collection, and the top hit is reported as the species. Additionally, a note is added indicating that this query did not produce an above-threshold score for any of the focal species.

### Performance of BMScan

We tested BMScan using a validation set consisting of all 17 focal species included within the BMScan reference collection as well as additional closely-related and sister species (Total = 15,503). The counts and sources per species of the genomes used in the testing set is shown in Table 4. BMScan successfully scanned all 15,503 query genomes and produced results in 16 min and 47 s using eight CPU threads. The results of this test are provided as supplement (Additional file 4). Overall, BMScan accurately identified species for 15,499/15,503 (99.97%) genomes using the Mash-derived threshold values. In terms of individual species, the tool correctly identified 5153/5155 (99.96%) of *E.coli* genomes, 7424/7426 (99.97%) of *Streptococcus pneumoniae* genomes, and 100% of the remaining focal species.

**Table 4:** Counts of genomes per species in validation set

Species	Counts		
	NCBI	PubMLST	Total
Neisseria meningitidis	792	0	792
Neisseria gonorrhoeae	393	0	393
Neisseria lactamica	7	135	142
Neisseria subflava	0	24	24
Neisseria mucosa	3	8	11
Neisseria polysaccharea	1	17	18
Neisseria cinerea	0	8	8
Neisseria elongata	4	1	5
Neisseria weaveri	0	1	1
Haemophilus influenzae	93	0	93
Haemophilus parainfluenzae	13	0	13
Haemophilus haemolyticus	5	0	5
Haemophilus parahaemolyticus	1	0	1
Streptococcus pneumoniae	7426	0	7426
Streptococcus mitis	58	0	58
Escherichia coli	5155	0	5155
Escherichia albertii	32	0	32
Escherichia fergusonii	7	0	7
Listeria monocytogenes	1313	0	1313
Listeria innocua	6	0	6
Total	15,309	194	15,503

The four genomes from the validation set that returned below-species threshold scores consisted of two *Escherichia coli* and two *Streptococcus pneumoniae.* Even though these four assemblies returned Mash scores that were below their respective species’ threshold, the top hit reported for them was the correct species. Furthermore, these assemblies were checked for quality, and it was identified that one *E.coli* genome had a genome size of 9.07mb, nearly twice as large as the median *E.coli* genome size, indicating a potential quality issue. Additional analyses can be performed on these assemblies to determine if they should be added to the reference collection, or if other factors contributed to their below-threshold score.

## Discussion and conclusion

Bacterial meningitis is a life-threatening infection that remains a serious global health concern. Identification of the bacterial meningitis pathogenic species is a critical step for successful treatment and response planning. The data provided by the genomic era has paved the way for the development of new methodologies and tools for advancing public health initiatives. Through these innovations, we have developed BMScan, a tool which incorporates the data from hundreds of thousands of whole-genome comparisons, along with curated species-specific thresholds, to rapidly and accurately identify the species of bacterial meningitis causing and closely-related pathogens.

Representative genomes for each of our 17 focal species were identified and compiled into a reference collection. Using the reference collection, we established species-specific Mash-score threshold values which assure high specificity for species assignment. Genomes that do not pass this similarity threshold, or pass the threshold for multiple species, will still have a presumptive species assignment and are flagged for more detailed examination. If BMScan returns the correct species as the top-hit with a lower similarity score than the threshold, this assembly should be both checked for quality control and for the potential that the sample represents an aspect of the diversity of the species that was not captured in the reference collection. If the latter is true, the genome can be added to the reference collection and new threshold values can be established for that species.

A major component of BMScan’s development involved the comparison of ANI and Mash. Our results corroborated those of Ondov et al. [10], indicating a strong correlation between ANI and 1-Mash for high-scoring comparisons (> 0.90). We also compared the run-time between ANI and Mash, and showed that Mash completes the same set of comparisons in a fraction of the time of ANI. Furthermore, we portrayed the resolution that Mash provides for species delineation, such that each species within *Neisseria* and *Haemophilus* in the reference collection formed distinct clusters.

The infrastructure of BMScan allows for streamlined updates by supplementing the reference collection with genomes for species of interest, running scripts which recalculate the Mash threshold values for that respective species, and modifying those values in the SQL DB. This ease of updateability enables BMScan to adapt efficiently by enabling the expansion of the tool to other species and the incorporation of novel strains for currently included species as we encounter them. BMScan serves as a proof of concept, and this framework of using whole-genome species-specific similarity threshold values with a reference collection for species identification could be extended for many other pathogens of interest.

BMScan can confidently assign species for thousands of bacterial meningitis causing and closely-related genomes on the magnitude of seconds or minutes, rather than hours or days. Furthermore, BMScan is easily updateable, allowing for both adaptability and maintainability. Overall, BMScan will be a core component in our pipelines for the analysis of bacterial meningitis pathogens.

## Availability and requirements

**Project Name**: BMScan

**Project home page**: https://bitbucket.org/ntopaz/bmscan

**Operating System**: Unix based operating systems such as Ubuntu, CentOS, Mac OSX, etc

**Programming Language**: Python

**Other requirements**: Python 3.4+, Mash 1.1+, SQLite3

**License**: GNU GPL

**Any restrictions to use by non-academics**: No license needed

## Additional files

Additional file 1: SQL database schema. (PNG 20 kb)

Additional file 2: Reference collection excel file with meta-data. (XLSX 41 kb)

Additional file 3: Bland Altman for ANIm and Mash. (PNG 120 kb)

Additional file 4: Results of validation test. (CSV 2265 kb)

Additional file 5: BioProject and PubMLST Ids for genome assemblies used in reference collection. (XLSX 17 kb)

## References

[1] Organization, W.H (1998). Control of epidemic meningococcal disease: WHO practical guidelines.

[2] World Health Organization, and Centers for Disease Control and Prevention. Laboratory methods for the diagnosis of meningitis caused by neisseria meningitidis, streptococcus pneumoniae, and haemophilus influenzae: WHO manual. 2011. http://scholar.google.com/scholar_lookup?title=Laboratory%20methods%20for%20the%20diagnosis%20of%20meningitis%20caused%20by%20Neisseria%20meningitidis%2C%20Streptococcus%20pneumoniae%2C%20and%20Haemophilus%20influenzae%0A%20%20%20%20%20%20%20%20%20%20%20%20%20%20%20%20%20%20%20%20%20%20%20%20%20%20%20&publication_year=2011.

[3] Thigpen MC (2011). Bacterial meningitis in the United States, 1998–2007. N Engl J Med.

[4] Wayne L (1987). Report of the ad hoc committee on reconciliation of approaches to bacterial systematics. Int J Syst Evol Microbiol.

[5] Konstantinidis KT, Tiedje JM (2005). Genomic insights that advance the species definition for prokaryotes. Proc Natl Acad Sci U S A.

[6] Richter M, Rosselló-Móra R (2009). Shifting the genomic gold standard for the prokaryotic species definition. Proc Natl Acad Sci.

[7] Goris J (2007). DNA–DNA hybridization values and their relationship to whole-genome sequence similarities. Int J Syst Evol Microbiol.

[8] Altschul SF (1997). Gapped BLAST and PSI-BLAST: a new generation of protein database search programs. Nucleic Acids Res.

[9] Kurtz S (2004). Versatile and open software for comparing large genomes. Genome Biol.

[10] Ondov BD (2016). Mash: fast genome and metagenome distance estimation using MinHash. Genome Biol.

[11] Bennett JS (2012). A genomic approach to bacterial taxonomy: an examination and proposed reclassification of species within the genus Neisseria. Microbiology.

[12] Bennett JS, Jolley KA, Maiden MC (2013). Genome sequence analyses show that Neisseria oralis is the same species as ‘Neisseria mucosa var. heidelbergensis’. Int J Syst Evol Microbiol.

[13] Leary O, Nuala A, et al. Reference sequence (RefSeq) database at NCBI: current status, taxonomic expansion, and functional annotation. Nucleic Acids Res. 2016;44(D1):D733-45. 10.1093/nar/gkv1189. Epub 2015 Nov 8. 10.1093/nar/gkv1189 PMC4702849 26553804

[14] Alexander S, Ison C (2005). Evaluation of commercial kits for the identification of Neisseria gonorrhoeae. J Med Microbiol.

[15] Vuong J (2016). Development of real-time PCR methods for the detection of bacterial meningitis pathogens without DNA extraction. PLoS One.

[16] Jorgensen JH, et al. Manual of Clinical Microbiology, Eleventh Edition: American Society of Microbiology; 2015. https://scholar.google.com/scholar?hl=en&as_sdt=0%2C11&q=manual+of+clinical+microbiology+jorgensen&btnG=.

[17] Retchless AC, et al. The Establishment and Diversification of Epidemic-Associated Serogroup W Meningococcus in the African Meningitis Belt, 1994 to 2012. mSphere. 2016;1(6). eCollection 2016 Nov-Dec. 10.1128/mSphere.00201-16 PMC5112335 27904879

[18] Bankevich A (2012). SPAdes: a new genome assembly algorithm and its applications to single-cell sequencing. J Comput Biol.

[19] Chin C-S (2013). Nonhybrid, finished microbial genome assemblies from long-read SMRT sequencing data. Nat Methods.

[20] Olm MR, et al. dRep: a tool for fast and accurate genomic comparisons that enables improved genome recovery from metagenomes through de-replication. ISME J. 2017;11(12):2864-2868. 10.1038/ismej.2017.126. Epub 2017 Jul 25. 10.1038/ismej.2017.126 PMC5702732 28742071

[21] Parks DH (2015). CheckM: assessing the quality of microbial genomes recovered from isolates, single cells, and metagenomes. Genome Res.

[22] Bland JM, Altman DG (1999). Measuring agreement in method comparison studies. Stat Methods Med Res.

[23] Saitou N, Nei M (1987). The neighbor-joining method: a new method for reconstructing phylogenetic trees. Mol Biol Evol.

[24] Cock PJ (2009). Biopython: freely available Python tools for computational molecular biology and bioinformatics. Bioinformatics.

[25] Talevich E (2012). Bio. Phylo: a unified toolkit for processing, analyzing and visualizing phylogenetic trees in Biopython. BMC bioinformatics.

[26] Letunic I, Bork P (2016). Interactive tree of life (iTOL) v3: an online tool for the display and annotation of phylogenetic and other trees. Nucleic Acids Res.

[27] Benson DA (1999). GenBank. Nucleic Acids Res.

[28] Jolley KA, Maiden MC (2010). BIGSdb: scalable analysis of bacterial genome variation at the population level. BMC Bioinformatics.

