# Supplementary figures and images for: BMScan: using whole genome similarity to rapidly and accurately identify bacterial meningitis causing species
Source: BMC Infect Dis. 2018 Aug 15;18:405. doi: 10.1186/s12879-018-3324-1 (PMC6094466; doi:10.1186/s12879-018-3324-1)

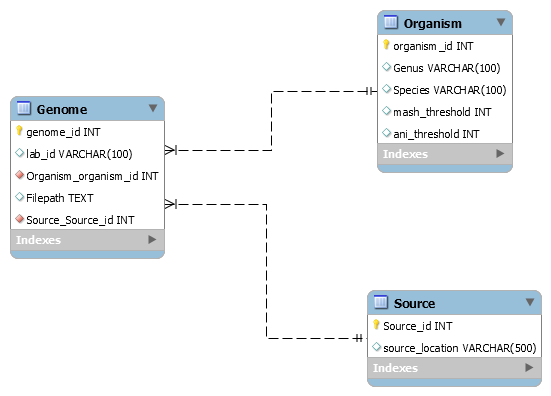

Supplement: Supplementary file 1 — SQL database schema. (PNG 20 kb) [file 12879_2018_3324_MOESM1_ESM.png]

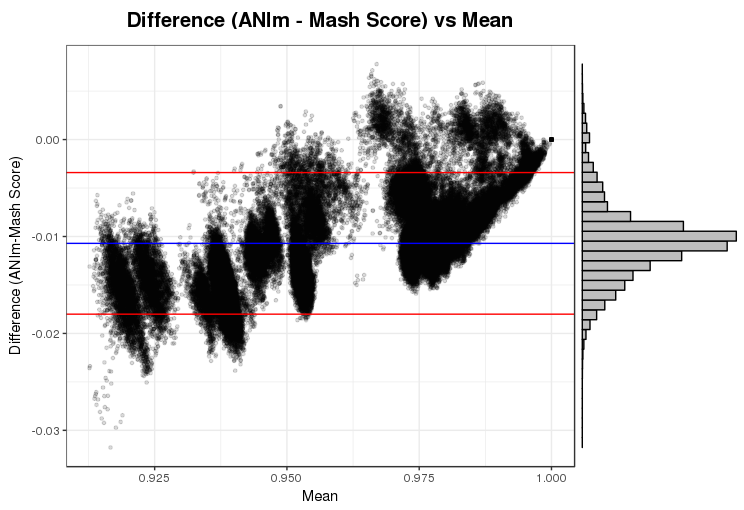

Supplement: Supplementary file 3 — Bland Altman for ANIm and Mash. (PNG 120 kb) [file 12879_2018_3324_MOESM3_ESM.png]
